# P-140. A Quality Improvement (QI) Project to Increase Chagas Disease Screening Rates in an At-Risk, Underserved Patient Population Living in Houston, Texas

**DOI:** 10.1093/ofid/ofae631.345

**Published:** 2025-01-29

**Authors:** Fernando H Centeno, Renatta Wetterman-Capo, Jaime Rueda, Eva Clark

**Affiliations:** Baylor College of Medicine, Houston, Texas; Baylor College of Medicine, Houston, Texas; Baylor College of Medicine, Houston, Texas; Baylor College of Medicine, Houston, Texas

## Abstract

**Background:**

Chagas disease (CD), caused by *Trypanosoma cruzi*, is a neglected disease of poverty affecting >6 million people in the Americas, including >200,000 in the US. While many are asymptomatic, 20-30% develop end-organ damage like Chagas cardiomyopathy. Early diagnosis and treatment are essential to reduce this risk; however, US clinicians generally are unaware of for whom CD screening is recommended (Table 1) and how to order it. >1.5 million people born in continental Latin America live in Harris County, TX, making this an important region in which to establish systematic CD screening. This QI project seeks to improve CD screening rates using electronic medical record (EMR) tools.

**Figure 1A:**
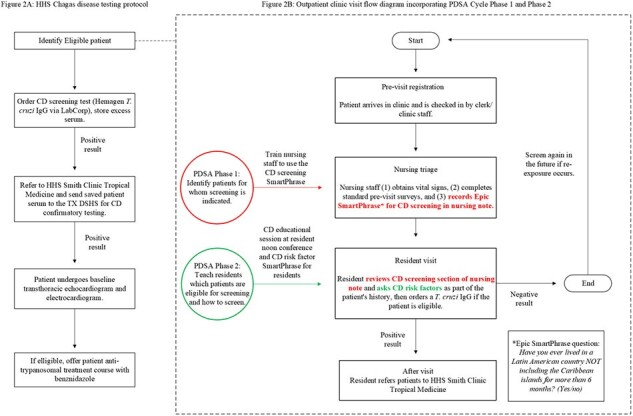
HHS Chagas disease testing protocol, Figure 1B: Outpatient clinic visit flow diagram incorporating PDSA Cycle Phase 1 and Phase 2

**Methods:**

This project is set in the Harris Health System (HHS), a public safety-net healthcare system serving >1 million outpatients annually; 52.9% are Hispanic and 45.9% are uninsured. Previous studies support a CD prevalence of ∼1%, suggesting that >5,000 outpatients/year present to HHS with ***undiagnosed*** CD. Our HHS CD screening protocol (Figure 1A) is based on US screening recommendations. We assembled a team of stakeholders and identified a HHS primary care clinic (MLK Clinic) as the pilot location. We used Epic Systems EMR tools to evaluate the patient population presenting to MLK Clinic over a 9 month period and develop corresponding QI interventions. The primary outcome is CD screening rate; see Table 2 for secondary outcomes.

**Figure 2:**
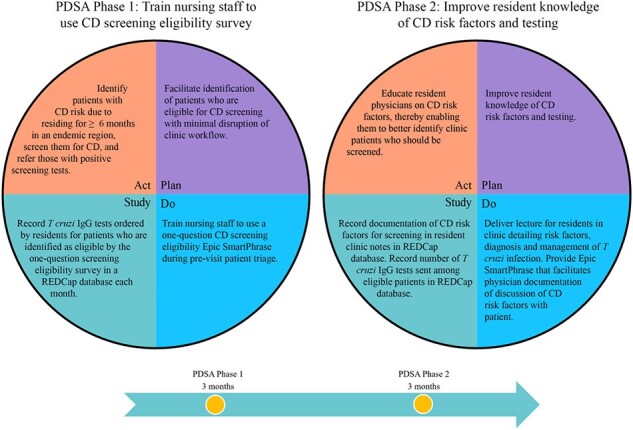
Plan, Do, Study, Act (PDSA) cycle structure

**Results:**

1,443 patients presented to MLK Clinic between 7/1/2023-4/30/2024, average age 26 years (range 0-95). A sample of 158 showed that 40% were eligible for CD screening based on birth country. Of those, only 12 *T. cruzi* IgG tests were ordered (2% of eligible patients). Together with stakeholders, we developed 2 interventions to improve CD screening rates (Figure 1B): 1) single-question Epic SmartPhrase used by nursing staff during patient triage and 2) physician CD educational session and Epic SmartPhrase. These will be applied in 2 PDSA cycle phases (Figure 2). We trained nursing staff on 4/2024 and began PDSA Phase 1 on 5/1/24.

**Conclusion:**

Early CD diagnosis and treatment reduce the risk of progression to devastating end-organ damage. This QI project will contribute to improved identification of at-risk patients through use of EMR tools for systematic screening.

**Disclosures:**

**All Authors**: No reported disclosures

